# Long-term benefits of omalizumab in a patient with severe non-allergic asthma

**DOI:** 10.1186/1710-1492-7-9

**Published:** 2011-05-24

**Authors:** Francesco Menzella, Roberto Piro, Nicola Facciolongo, Claudia Castagnetti, Anna Simonazzi, Luigi Zucchi

**Affiliations:** 1 Department of Respiratory Diseases, Santa Maria Nuova Hospital, Reggio, Emilia, Italy

## Abstract

**Introduction:**

Currently, omalizumab is indicated for the treatment of patients with severe allergic uncontrolled asthma despite optimal therapy.

**Case presentation:**

We studied a 52-year-old man who has been suffering from severe non allergic steroid-resistant asthma with increased levels of total IgE and a lot of comorbidity. After a 3 years long treatment with omalizumab, he presented a significant improvement in disease control in terms of hospitalizations, exacerbation, quality of life and lung function with good safety profile.

**Conclusion:**

Our case shows, after a long follow-up, how omalizumab can be effective in a severe form of non-atopic asthma. It is therefore hoped that further studies can identify indicators that are able to give to clinicians information about patients who can be responsive to monoclonal anti-IgE antibody even if non allergic.

## Introduction

Patients with severe asthma often have a poor control of their disease; they represent the subgroup that absorbs most of the costs [1,2]; for these reasons it has given rise to the need to get new drugs able to improve control. The only biological drug available for the treatment of severe asthma is omalizumab. A number of clinical trials have been performed in order to evaluate either the efficacy or the safety of the above-mentioned drug: the results showed that this molecule is able to significantly improve asthma control and quality of life, with an excellent safety profile [3-5].

Actually, as set by the European Medicine Agency (EMA) [6] and GINA Guidelines [7], in Europe omalizumab is indicated as an add-on therapy aimed at improving asthma control in adult and adolescent patients (12 years of age and above) with severe persistent allergic asthma who have a positive skin test or in vitro reactivity to a perennial aeroallergen and who show reduced lung function (FEV1 <80%) as well as frequent daytime symptoms or night-time awakenings and who have had multiple documented severe asthma exacerbations despite daily high-dose inhaled corticosteroids, plus a long-acting inhaled beta2-agonist. This treatment option is limited to patients with baseline IgE level of 30 to 1.500 IU/ml and body weight of 20 to 150 kg.

In this study we describe the case of a man suffering from severe non-allergic steroid-resistant asthma associated with important comorbidity in which omalizumab induced an extraordinary improvement of symptoms, health-related quality of life (HRQoL), exacerbations and lung function.

## Case Report

In May 2006 a Caucasian 52-year-old man came to our observation because of severe persistent asthma not controlled despite an extensive therapy (formoterol 18 mcg/day; budesonide 640 mcg/day; tiotropium bromide 18 mcg/day), oral steroids (prednisone 25 mg/day). The patient has a history of 2 severe exacerbations with hospitalization, several mild and moderate exacerbations (4-5/year) treated with increase of systemic steroids, frequent nocturnal awakenings (2-3 per night) and daily use of salbutamol as rescue medication (3-4 times/day) [7], frequent nocturnal awakenings (2-3 per night) and daily use of albuterol as rescue medication (3-4 times/day) with side effects related to inhaled steroid (oral candidiasis) and LABA (tachycardia) that had prevented him from increasing the dosage of these drugs.

He was a former smoker (9 pack-year), asthma and rhinitis were diagnosed in 1991 and he was also affected by nasal polyps, hypertension, diverticulosis of the colon, moderate obesity, dyslipidemia and lactose intolerance. He was hospitalized in the Respiratory Department because of asthma exacerbation in 2004 and 2005. The patient was not sensitized to aero- and food allergens and his respiratory symptoms were not affected by seasons. Vesicular breath sounds were markedly reduced and wheezes were present. Lung function tests showed a severe obstruction: FEV1 1.52 (46% of predicted) and FEV1/FVC 0.41 [Table 1]. The ventilatory defect showed reversibility (23%) after albuterol administration. A high-resolution chest CT showed no signs of parenchymal lung disease. Blood tests showed peripheral eosinophilia (8%) and total IgE were 272.6 KIU/L without specific IgE to inhalant or food allergens testing with ImmunoCap (Phadia, Sweden).

**Table 1:** Omalizumab treatment effectiveness

Outcomes	Basal	Basal (post beta agonist)	32-week	3-years	3-years (post beta agonist)
GETE rating			Excellent	Excellent	
AQLQ (median, range)	1.71		3.23	5.43	
FEV1 %	46%	60%	40%	53%	71%
FEV1/FVC	0.49	0.49	0.46	0.49	0.57
FEF25%	25%	30%	12%	26%	33%
FEF50%	15%	18%	13%	18%	22%
FEF75%	16%	14%	13%	13%	15%
Hospitalization	2		0	0	
Exacerbation mild + moderate	5 for year		0	2 (-60%)	
IgE	272.6 IU/ml			419 IU/ml	

Even the skin prick test for common aeroallergens was negative. The allergens we performed (both for cutaneous and serological tests) were: grasses, parietaria officinalis, ragweed, mugwort, plantain, birch, cypress, walnut, dust mites, molds (Aspergillus Fumigatus, Alternaria, Cladosporium, Penicillium), cat and dog epithelium.

We used the Asthma Quality of Life Questionnaire (AQLQ) [8] to assess the patient's QoL with an initial score of 1,71 points, indicating a poor HRQoL [Table 1]. Given the poor asthma control, the severe obstruction and total IgE, we hypothesized that treatment with omalizumab could be effective, despite the absence of sensitization to inhalant allergens.

The patient signed the informed consent and started the treatment in July 2006 (300 mg every 15 days subcutaneously). An improvement in symptoms of asthma control was evident after only 16 weeks of treatment; the patient had no exacerbation. The lung function parameters were essentially unchanged but the AQLQ score increased to 3.23 [Table 1].

In order to evaluate the efficacy of omalizumab, the Global Evaluation of Treatment Effectiveness scales (GETE) [3,9,10] was used and the result was good. After 32 weeks of treatment the discontinuation of systemic steroid and tiotropium was possible. In that interval, there had been no exacerbations; spirometry showed a slight worsening (FEV1 1.33 l), while the AQLQ score was further improved (4.62). The GETE was excellent. The patient was followed until July 2010 and in that range he had only two mild relapses and no hospitalization. The AQLQ score arrived at 5.43 confirming a marked improvement in the quality of life; spirometry showed a discrete increase (FEV1 1.70 l; 53% of predicted) compared to baseline (FEV1 +15%). The GETE was confirmed excellent [Table 1].

Finally, the assay of serum IgE at the end of follow-up was 419 IU/ml, with an increase compared to baseline.

## Discussion

According to current guidelines, omalizumab is a safe and effective add-on treatment which, in suitable patients [7], allows them to obtain better control of asthma by reducing the number of exacerbations and the use of steroids and improving the quality of life [3,4].

Actually, one only report [11] concerns the effectiveness of this drug in a patient with non-allergic asthma (who had high total IgE). Several authors showed that the different phenotypes of asthma have several likeness, with similar cytokine and cellular patterns both in allergic and non-allergic asthma [12,13]. Therefore, IgE may have a key role in the inflammatory cascade (even in the absence of a proved aeroallergen) contributing to bronchial hyper reactivity and remodeling in asthma. Also, it is well known that higher values of IgE are associated with higher hyper reactivity and more severe obstruction [14].

However, it was shown that the block of free IgE is not the only pharmacological effect of omalizumab, as it also promotes the down-regulation of the expression of the high-affinity receptor FcεRI, causing a further reduction of IgE on the cell surface [14,15].

Also, a study by Berger and cohautors showed that the in vitro incubation with omalizumab of bronchial tissue from asthmatic patients, inhibits specific and aspecific bronchial hyper-responsiveness. This effect should be related to the inhibition of bronchial mast-cells degranulation [16].

Based on these considerations, we hypothesized that treatment with omalizumab in our patient could be effective, in spite of what is indicated by the guidelines and the manufacturer.

The clinical and instrumental data show a significant improvement in disease control in terms of hospitalizations and exacerbations. In order to evaluate the effectiveness at 32 weeks and at 3 years, we used the Global Evaluation of Treatment Effectiveness scale (GETE) [3,9,10]. The evaluation is performed independently by both investigator and patient using the same 5 point scale. This scale ranges from excellent through good, moderate, and poor to worsening. A good or excellent response is suggested as a means of defining a patient who has responded to treatment. In our patient the GETE rating at 3 years was excellent, confirming the effectiveness of omalizumab.

Health-related quality of life (HRQoL) was assessed by means of the AQLQ score [8]. The AQLQ is composed of 32 questions which cover four domains: activity limitation, symptoms, environmental stimuli and emotional function. Subjects recall their experiences during the previous 2 weeks and score a number of asthma-related problems on a 7-point scale from 1 (maximum impairment) to 7 (no impairment). We used an overall summary index, which is the mean of the responses to the 32 items (total AQLQ score). The AQLQ was found to be valid, reproducible and responsive to change over time and a change in questionnaire score of 0.5 or more points has been determined to be the minimal clinically important difference [8]. In our patient the quality of life improved significantly, with the AQLQ score progressively increasing over time. The lung function parameters were stable during the first months, showing a rise after four years. This could be explained by the bronchial remodelling caused by the long-standing ashtma, which needed several months of therapy to appreciate an improvement.

In our study, it is possible that specific allergic sensitivity was simply not identified, as the range of potential allergenic agents is considerably larger than current diagnostic reagents can address, so false-negative allergen-skin-test results are likely to happen.

Also, in this patient the contribution of placebo effects cannot be excluded. However, improvements were seen in objective parameters such as lung function and number of exacerbations as well as symptom improvement.

The possibility that the improvement is related to a greater surveillance is unlikely as it was previously followed consistently with good compliance. In addition, the timing are closely related with the administration of omalizumab.

About the increase of total IgE, it is well-known that patients treated with omalizumab may exhibit reduction of serum free IgE levels with increased total IgE due to the formation of IgE anti-IgE small immune complexes, which have a longer half-life than free IgE [17,18]. However, this increase has not pathological significance.

In conclusion, our work shows after a long follow-up the effectiveness of omalizumab with a good safety profile in a severe form of non-atopic asthma with increased levels of total IgE. It is therefore hoped that further studies identify new indicators that can give to clinicians information about asthmatic patients who can be responsive to monoclonal anti-IgE antibody even if non allergic.

## Consent

Written informed consent was obtained from the patient for publication of this case report.

## Abbreviations

AQLQ: Asthma Quality of Life Questionnaire; FEV1: Forced Expiratory Volume in 1 Second; FVC: Forced Ventilatory Capacity; GETE: Global Evaluation of Treatment Effectiveness; GINA: Global Initiative for Asthma; IgE: Immunoglobulin E; QoL: Quality of Life

## Competing interests

FM participated in clinical trial for Novartis and received travel sponsorship from Novartis, Astra-Zeneca e Glaxo Smith-Kline. NF received travel sponsorship from Astra-Zeneca, Pfizer, Boehringer. CC received travel sponsorship from Nycomed, Astra-Zeneca. LZ received travel grant from Novartis, Astra-Zeneca, Glaxo Smith- Kline and participated in contracted research for Novartis, Glaxo Smith-Kline, Boehringer-Ingelheim.

## Authors' contributions

FM coordinated diagnostic and therapeutic stages and was one of the principal contributors in writing the manuscript. RP contributed to the clinical approach, analyzed and interpreted the data and was a major contributor in writing the manuscript. NF was a contributor in writing the manuscript. CC was a contributor in writing the manuscript. AS was a contributor in writing the manuscript. LZ was a contributor in writing the manuscript and he gave final approval of the version to be published. All authors read and approved the final manuscript.

## Authors' information

The Centre the authors belong to participated in the last International Clinical Trial on omalizumab (CIGE025A2425) as National Coordinating Centre for Italy.
